# Oxidation-Induced Anisotropic Subsurface Segregation in SnTe Nanowires

**DOI:** 10.1021/acs.chemmater.5c03523

**Published:** 2026-07-28

**Authors:** Dorota Janaszko, Piotr Dziawa, Jakub Polaczyński, Anna Kaleta, Bogusława Kurowska, Marta Bilska, Jȩdrzej Korczak, Aleksandr Kazakov, Tomasz Wojciechowski, Jakub Turczyński, Jerzy Da̧browski, Janusz Sadowski, Sławomir Kret

**Affiliations:** † 86906 Institute of Physics, Polish Academy of Sciences, Al. Lotników 32/46, PL-02668 Warsaw, Poland; ‡ International Research Center MagTop, Institute of Physics, Polish Academy of Sciences, Al. Lotników 32/46, PL-02668 Warsaw, Poland; § Institute of Experimental Physics, Faculty of Physics, University of Warsaw, ul. Pasteura 5, PL-02093 Warsaw, Poland; ∥ Ensemble3 Center of Excellence, ul. Wólczyńska 133, PL-01919 Warsaw, Poland

## Abstract

In this study, we present the results of structural investigations of topological crystalline insulator (TCI) SnTe nanowires (NWs) grown by two different methods: autocatalytic and gold-assisted. The growth was performed using two distinct approaches: molecular beam epitaxy (MBE) and physical vapor deposition (PVD). Cross-sectional studies of oxidized NWs reveal a pronounced, unusual, anisotropic elemental distribution and a complex oxidation behavior. Taking into account the differences in the diffusion coefficients of the constituent elements and the high concentration of intrinsic cation vacancies, a two-step mechanism has been proposed. The initial step involves the formation of a thin surface oxide layer based on the Cabrera-Mott model adapted for semiconductors. This is followed by interdiffusion, described as the Kirkendall effect, in which tin migrates outward, whereas oxygen migrates inward into the NW. The process is not entirely uniform, and throughout the formation of the amorphous oxide skin, crystalline filaments are carved out as a result of ion migration. Concurrently, filaments act as channels, facilitating ion exchange through the associated vacancy diffusion mechanism. For Au-catalyzed NWs, thin Au films in the form of a ferrule extending several tens of nanometers were observed beneath the nanodroplets along the sidewalls, effectively suppressing oxygen adsorption in these regions. Chemical analysis of oxidized NWs coated with a Ti/Au bilayer, commonly employed for electrical contacts, shows that Ti binds oxygen from the oxide. However, this does not alter the previously created chemical segregation. The results clarify and advance the understanding of interface stability and oxidation in IV–VI semiconductor nanostructures, offering guidance for device integration.

## Graphical abstract

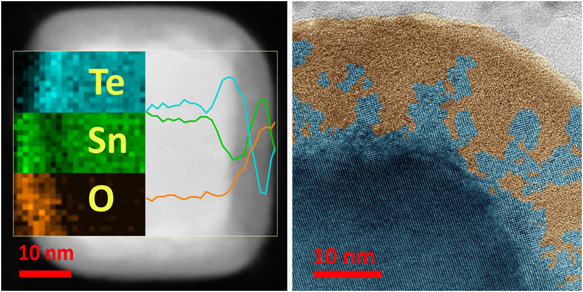

## Introduction

Topological crystalline insulators have attracted considerable attention in the past decade due to the presence of topological surface states (TSS) that exhibit linear dispersion and a helical spin texture. Narrow-gap IV–VI semiconductor binary compounds and their substitutional solid solutions are the first theoretically predicted^1,2^ and experimentally confirmed^3–5^ TCI materials. Unlike conventional topological insulators (TIs) protected by time reversal symmetry, in TCIs the TSS are protected by space group symmetries. The transition from the trivial state to the TCI state can be controlled by altering the chemical composition,^6,7^ temperature,^8^ hydrostatic pressure,^9^ or lattice distortion.^10^

One of the TSS probing methods relies on measuring magneto–transport features. In contrast to bulk crystals, the TSS states are expected to be more pronounced in the NWs due to the increased surface-to-volume ratio. Notably, previous studies on SnTe NWs have already reported weak antilocalization and Aharonov-Bohm oscillations, while a nontrivial Berry phase was derived from the analysis of Shubnikov-de Haas oscillations. Those magneto-transport phenomena are often regarded as signatures of Dirac quasiparticles.^11,12^ This paves the way for the exploitation of various properties of SnTe nanostructures: from thermoelectric properties,^13^ interactions of correlated electronic states (ferroelectricity, superconductivity),^14^ to exotic TSS with spin–momentum locking for spintronics and quantum computing.^15^

Experimental studies of the electron transport properties in SnTe NWs are hampered by the oxidation process. This issue is more extensively discussed in the Supporting Information Part 1. The reasons for this behavior are surface degradation and modification, which can strongly affect the properties of the TSS. Investigations of bulk SnTe oxidation processes revealed the formation of different species near the surface. The inhomogeneous distribution of tin and tellurium oxides was found.^16^ In the case of Bi_2_Se_3_ TI, the oxides were shown to reduce the mobility of the electron gas at the surface.^17^ Surprisingly, despite the great importance of surface quality for TI and TCI properties, experimental reports on this topic are scarce.^16–18^

To obtain more information about the surface properties of SnTe nanowires, structural studies are conducted using a transmission electron microscope (TEM). The results are presented for SnTe nanowires produced by two different methods: MBE and PVD. Oxidation results in the formation of a core–shell-like structure, where a crystalline NW interior is surrounded by an amorphous skin composed of different chemical fractions. We explain the observed redistribution of elements by the Kirkendall effect. Differences in diffusion coefficients and atomic mobility are the driving force that leads to the migration of atoms. In SnTe nanowires, the Kirkendall mechanism is possible due to oxidation and the presence of native vacancies. In addition, the effect of metallization on the surface of oxidized NW is investigated.

## Experimental Section

### Growth of the Nanowires

The investigated SnTe NWs were grown by two methods. The MBE technique was applied to the growth on (100)- and (111)-oriented silicon substrates (Azelis). The growth was carried out using a binary solid source of SnTe, which was obtained by chemical synthesis of elemental Sn and Te of 4N85 and 5N+ metal basis purity, respectively. The beam equivalent pressure (BEP) of the molecular beam ranged from 4.5 × 10^–8^ mbar to 5.0 × 10^–7^ mbar. The growth process was monitored using reflection high-energy electron diffraction (RHEED). The nanowires were grown by the vapor–liquid–solid (VLS) mechanism^19^ in the temperature range of 400 to 440 °C. Monodispersed 20 nm water-based colloidal Au nanoparticles (Ted Pella, Inc.) were used as a metal catalyst.

The second set of NWs was grown using physical vapor deposition. The growth was carried out in a single-zone horizontal tube furnace in a quartz ampule preheated to 600 °C. As a source material, a polycrystalline SnTe ingot crumbled into smaller pieces was used, with the same purity level as that used for the MBE growth. Si(100) substrates were covered with a 1.8 nm-thick Au film deposited by thermal evaporation in a separate process. The ampule containing the SnTe source and the substrates was pumped down to a pressure of 10^–5^ mbar and sealed. Catalyst nanodroplets on the surface were created at the beginning of the process by placing the substrate at a temperature higher than the Au–Si eutectic point temperature. The positions of the source material and the substrates in the ampule were chosen to provide temperatures of approximately 600 and 440 °C, respectively, to ensure sufficient molecules flow from the source to the substrate. After the growth, the samples were cooled down naturally when the ampule was removed from the furnace.

Two types of samples were prepared for the studies: Au-assisted (MBE- and PVD-grown) and autocatalytic (MBE-grown) nanowires. All the samples were stored at room temperature under normal oxygen and humidity conditions for 3 days. The process of free unintentional oxidation was initiated when the sample was removed from the vacuum, specifically from the MBE system or the ampule, respectively. Additionally, the PVD-grown NWs were used to study the effects of an additional coating. They were mechanically transferred onto a separate Si(100) substrate and covered by thermal evaporation with a 10 nm-thick Ti followed by a 100 nm-thick Au—a bilayer commonly used to fabricate contacts for transport measurements.

### Microscopic Investigations

All examinations were carried out at room temperature. Scanning electron microscopy (SEM) studies were performed using a ZEISS Auriga CrossBeam microscope. For deeper insight into the structure of NWs, a FEI-Titan 80–300 TEM operating at 300 kV was used, equipped with a spherical image aberration corrector and scanning transmission electron microscopy (STEM) high-angle annular dark-field (HAADF) detector. The range of collection angles and the corresponding camera lengths used during the investigations are listed in the Supporting Information Part 2 (see Table S1). To facilitate the preparation of TEM samples, the NWs were mechanically transferred onto a copper grid with a holey carbon film, without the use of a liquid buffer. The lamellas for the cross-section studies were prepared using Thermo Scientific’s Helios G1 NanoLab DualBeam 600 SEM with a FIB lift-off procedure and Omni-Probe nanomanipulator.

The distribution of elements and semiquantitative estimation of local chemical composition were investigated by energy dispersive X-ray spectroscopy (EDS) using the EDAX system at energies up to 15 keV. The total relative error for EDS quantification is 5%.^20^ The EDS spectra on the NWs cross-section were collected along the specific zone axis.

TEM Imaging & Analysis (TIA) FEI software routines were implemented for elemental concentration distributions, followed by data processing. Three-dimensional images of the spectra were analyzed using the SiTools software plug-in.^21^ In such a data set, each pixel of the mapped area contains the full spectrum.

## Results and Discussion

Tin and lead tellurides and solid solutions based on them crystallize in a rock-salt structure. This is explained by ab initio modeling of the relaxed structure.^22^ This also applies to the NWs studied here, as presented later in this work. Accordingly, all NWs have a rectangular cross-section. Nevertheless, as shown in the recent paper, an unusual 5-fold cross-section can be obtained for a ternary Pb_1–*x*_Sn_*x*_Te substitutional solid solution. However, such a structure is still formed by segments with a rock-salt crystal structure.^22^ Regardless of the deviation from the normal to the substrate surface, the analyzed NWs show a [001] growth direction. This is a direct consequence of the natural (001) growth plane in IV–VI telluride NWs.^23^ The crystal structure of the grown SnTe NWs is uniform, and no structural defects such as stacking faults known from III–V or II–VI NWs are observed (see also^24^).

As shown in Figure 1a–c, all the MBE- and PVD-grown NWs are randomly distributed on the substrate surface. The surface density of the nanowires ranges from 10^7^ to 10^9^ cm^–2^, for both growth techniques. In the case of Au-catalyzed NWs, it can be controlled by the density of catalyst nanodroplets, which is strongly dependent on the thickness of the initially deposited Au layer or the dispersion of colloidal Au nanoparticles. The typical growth rate of the MBE-grown NWs reported here is 1 μm/h, and their width is less than 100 nm. SEM images show that the autocatalyzed MBE-grown NWs grow out-of-plane, but do not have a well-defined orientation relative to the substrate surface when compared to those Au-catalyzed (Figure 1a,b). In the case of PVD-grown samples, irregularly shaped crystallites are visible apart from NWs, as revealed in Figure 1c. Some of them are attached to the Au catalyst droplet that is larger than those usually observed at the NW tip (<100 nm). The length of the PVD-grown NWs ranges from 0.8 to 12 μm, and the transversal dimensions range from 200 to 50 nm, respectively.

**Figure 1:**
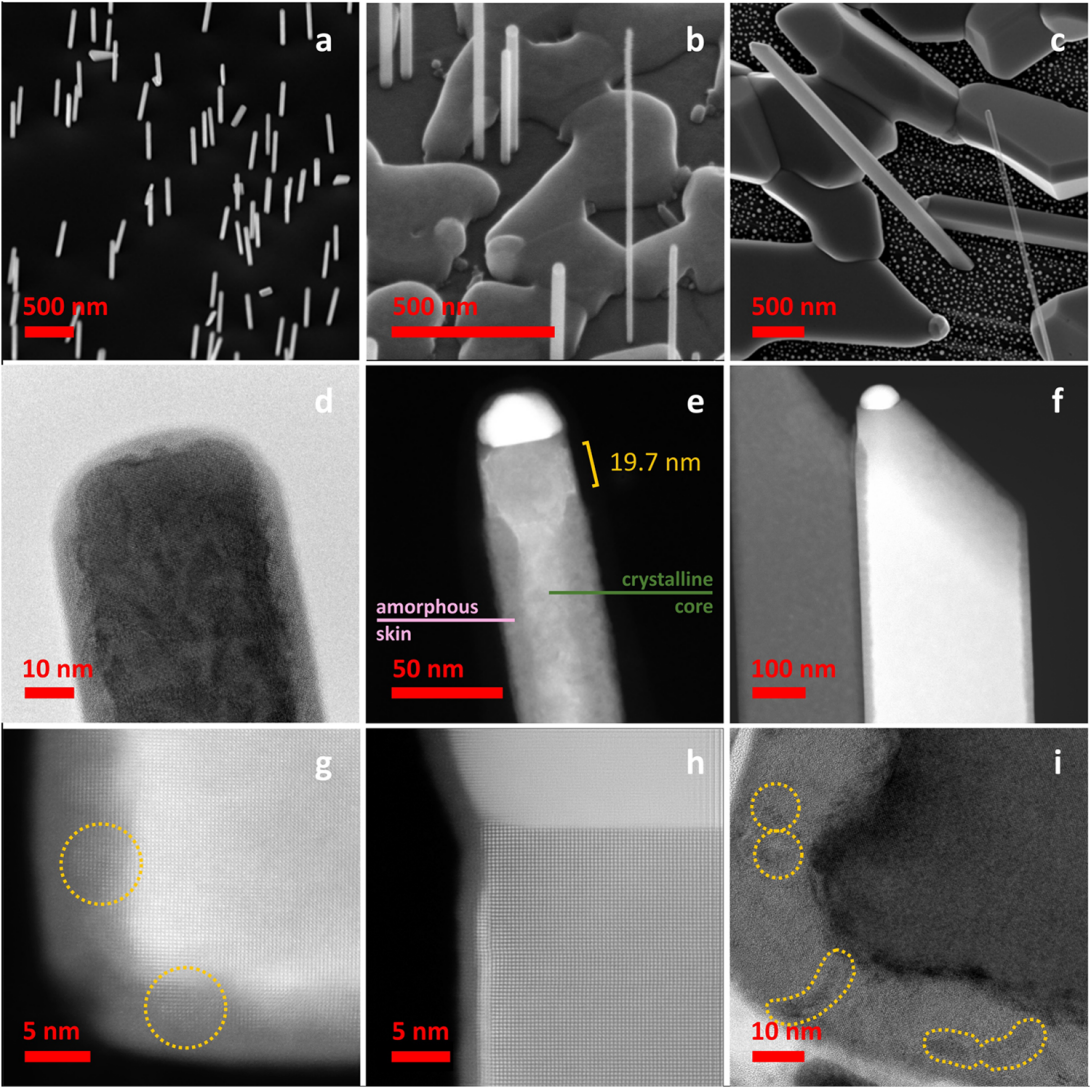
Microscopic images of the investigated NWs. (a–c) SEM images of (in order) autocatalyzed MBE-grown, Au-catalyzed MBE-grown, and Au-catalyzed PVD-grown nanowires. Tips of nanowires: (d) TEM image of autocatalyzed NW, (e, f) HAADF-STEM images of Au-catalyzed MBE-grown and PVD-grown nanowires, respectively. (g, h) HAADF-STEM images and (i) HRTEM image showing a close-up of the structural features of interest. More details on the filaments and precipitates are included in Supporting Information Part 2.

When comparing the tips of NWs (Figure 1d–f), it can be seen that, in addition to the regularly shaped spherical catalyst nanodroplet at the top of the Au-catalyzed NW, a distinct feature is consistently observed - a thin film of gold at the NW sidewalls below the nanodroplet (Figure 1e and f). Such an observation was published previously by Sadowski et al.^24^ It forms a ferrule surrounding the NW, typically several tens of nanometers long, and protects the NW from oxidation in the outer region. HAADF-STEM images show that such films are not connected to catalyst droplets (e.g., Figure 1h). For more details on this subject, see Supporting Information Part 2.

The unprotected NW sidewalls have an amorphous oxide skin with a smooth outer surface, while the interface with the crystalline interior is rough. Observations of Au-catalyzed NWs reveal that such a skin tends to be thicker in PVD-grown NWs than in the MBE-grown ones (compare, e.g., Figure 1g and i). We attribute this to the difference in the growth mechanism between the two techniques. Due to the dissimilarity in the substrate cooling method and environment, atomic-scale surface roughness is more pronounced in the PVD growth method. Residual particles present during the cooling process are incorporated into the sidewalls through direct deposition from the environment, rather than by the VLS mechanism. This is not the case in the MBE technique, where the growth is stopped by cutting off the molecular fluxes, and background pressure does not influence the growth process.

Interestingly, crystalline inclusions are immersed in the amorphous phase. Depending on the amorphous skin thickness, protrusions (in thinner, Figure 1g), or long filaments and precipitates (in thicker, Figure 1i) occur. Although all these inclusions share the same (rock-salt) crystal structure with the NW interior, their orientation may differ. This is evident in the filaments. They are connected to the NW interior, but their parts are twisted compared to it within ±5°. For more information on this, see Supporting Information Part 2.

The EDS profiles (normalized to 100%) collected in the area containing the gold ferrule reveal that the maximum Au content corresponds to approximately 4 at. % at this location (Figure 2a). The thickness of such a film, and thus the degree of protection it provides, is not fixed. It shows local discontinuities that cause oxidation. In the areas where the Au concentration decreases, the width of the NW increases, as shown in Figure 2b. We do not yet know what caused such a significant gap in the ferule (coverage close to or equal to 0%). The enlarged thickness of the NWs caused by oxidation shows that the migration of the tin atoms toward the oxygen and outward from the initial volume of the NW is apparent. In the areas where gold is present, the degradation of the structure does not occur. In the case of autocatalytic NWs, the amorphous skin appears homogeneously on the entire surface (see Figure 2c). It is interesting to note that the oxide thickness is significantly smaller for autocatalytic nanowires than for the Au-catalyzed ones (approximately twice as small).

**Figure 2:**
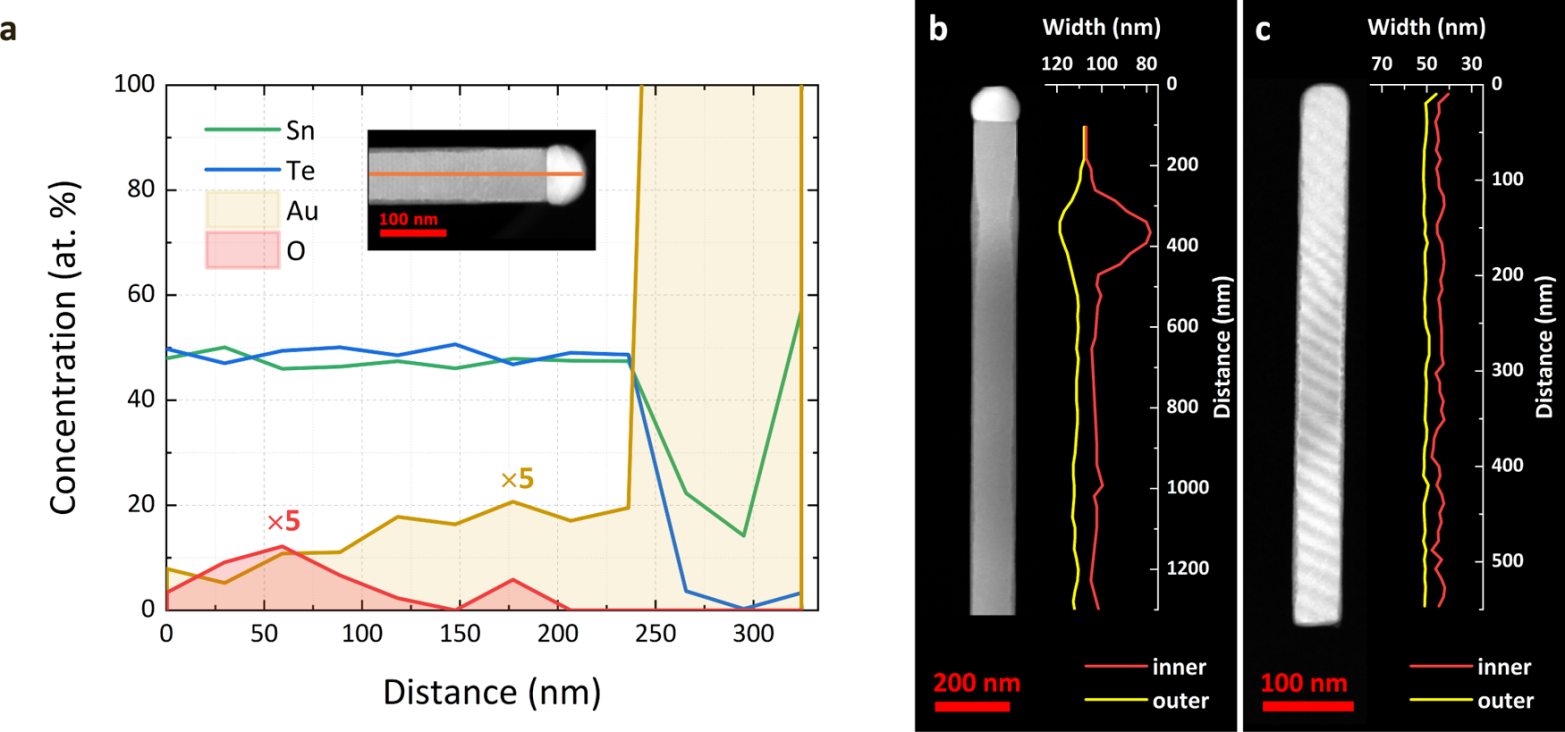
(a) EDS profile of the elemental composition in the MBE-grown NW collected along the orange line shown on the HAADF-STEM inset. The concentration of gold and oxygen is multiplied by 5 for better visibility. HAADF-STEM images of the MBE-grown NWs: (b) Au-assisted and (c) autocatalytic. The insets show the outer and inner widths of the nanowires, the latter referring to the crystalline interior of the nanowires. The fringes visible on the autocatalytic nanowire (c) are a moiré pattern.

To investigate the extent of the inward oxidation and chemical composition changes, the EDS profiles (Figure 3a,b) and elemental mapping (Figure 3c,d) were performed. The profile from the cross-section shows a symmetrical distribution of the studied elements, with no essential deviations. An interesting observation is the radial migration of elements. Analysis reveals a region approximately 5 nm thick, depleted in Sn, located beneath the outermost part of the amorphous skin, which is identified as tin oxide. Due to the uniform oxidation process on all sidewalls, a resulting layered structure reproduces the symmetry of the square-shaped NW cross-section, which is explicit in the elemental maps. The consistently low concentration of oxygen (less than 10 atom %) in the area inside the NW in the profile in Figure 3b does not originate from the incorporation of oxygen during the growth process. This trace is related to oxidation during transport between microscopes under ambient conditions.

**Figure 3:**
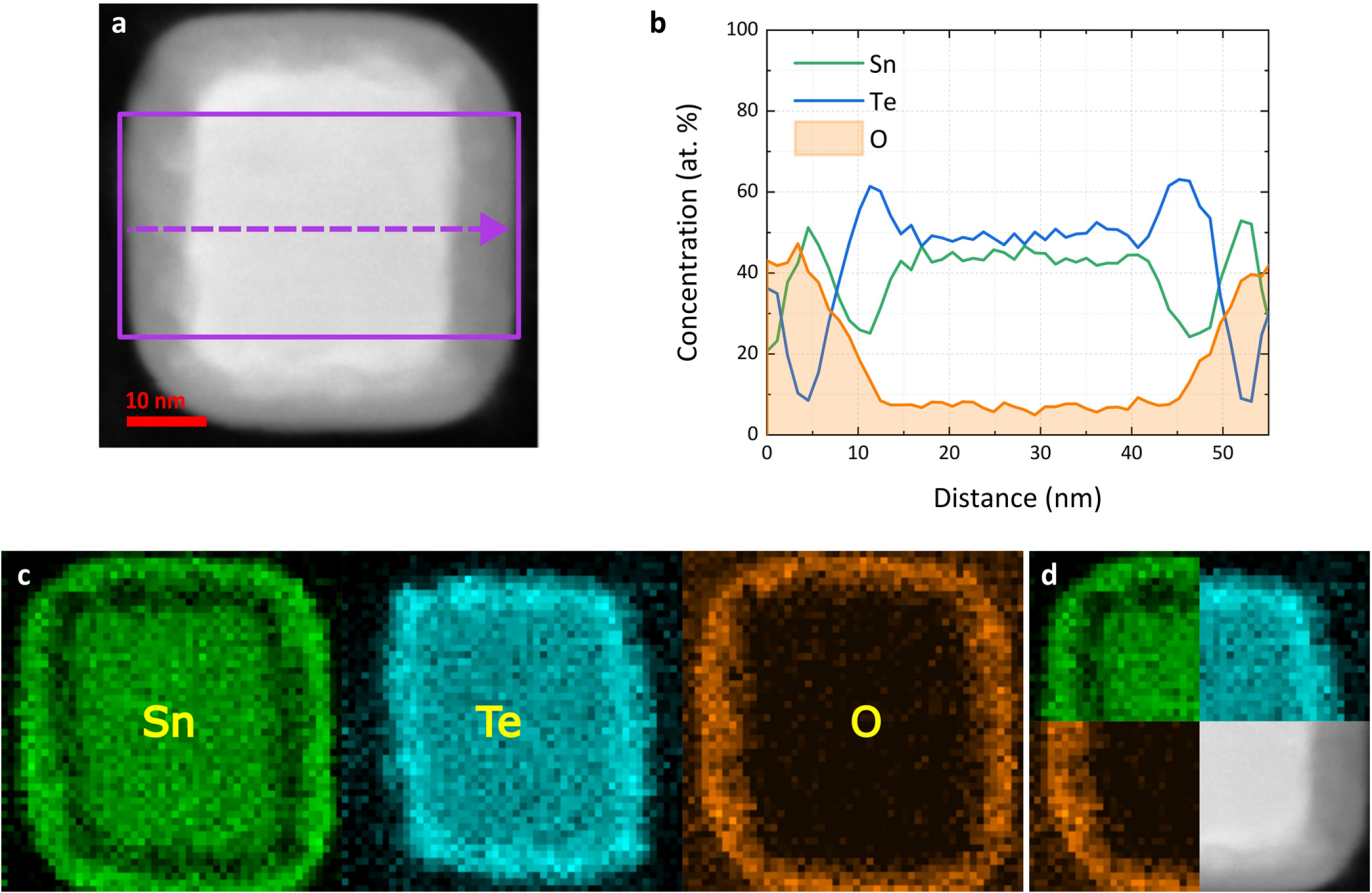
(a) HAADF-STEM image of the MBE-grown autocatalytic NW cross-section along the [100] zone axis. (b) EDS profiles of the average elemental concentration integrated along the dashed line from the purple rectangle in panel (a). The atomic concentration of the studied elements is normalized to 100 atom %. Values at both ends of the profile (approximately 3 nm thick each) are overestimated due to experimental conditions. (c) EDS elemental net intensity maps for Sn, Te, and O taken from the cross-section shown in panel (a). The intensity of the Te elemental map is doubled to enhance the visibility of the distribution. The maps are slightly tilted due to drift in the TEM microscope. (d) A combination of elemental EDS maps and the HAADF-STEM image.

Based on the elemental distribution, presented in Figure 3, two main regions in the amorphous skin can be distinguished. The outermost one is rich in tin and oxygen. The intensity ratio in the profile suggests in this area the presence of mainly SnO_*x*_ closer to monoxide instead of the more stable SnO_2_. Below this region, a significant accumulation of tellurium (20% more than in the NW interior) is present. From the composition profile, the estimated Sn:Te ratio defines three regions: (i) the NW interior with a composition close to stoichiometric (47:53, with a slight excess of Te expected in SnTe), (ii) the Sn-poor ring (about 30:70), and (iii) the Sn-rich outer region (about 85:15).

As mentioned before, magnetotransport studies belong to one of the important techniques used to investigate the properties of TSS in SnTe NWs. This requires the fabrication of metallic contacts attached to a single NW. To study the interface between the oxide skin and metallic contact, we coated some of the PVD-grown NWs with a Ti/Au bilayer, often used to fabricate contacts. Figure 4a presents profiles of the constituent elements of the nanowire, oxygen, and metallization bilayer. The average Sn:Te ratio in the interior region of the NW shows relatively constant and close to a stoichiometric composition. The displacement of elements at the edge is not as spectacular as in the case of nanowires fabricated by the MBE method. However, the elemental composition in the tin-depleted area resembles the behavior observed in the previous case. The less pronounced inversion of the Sn:Te ratio in this region is related to the thickness of the oxide skin, higher than in the MBE-grown NWs, and a more developed interface between this skin and the crystalline nanowire interior. Noticeably, the portion of oxygen present in the skin penetrates the titanium layer. The polycrystalline gold layer has not been studied because it is not involved in the migration processes. Due to the same storage environment and transport method, the PVD-grown NWs also exhibit a low trace of oxygen inside the NW, as observed in the cross-section.

**Figure 4:**
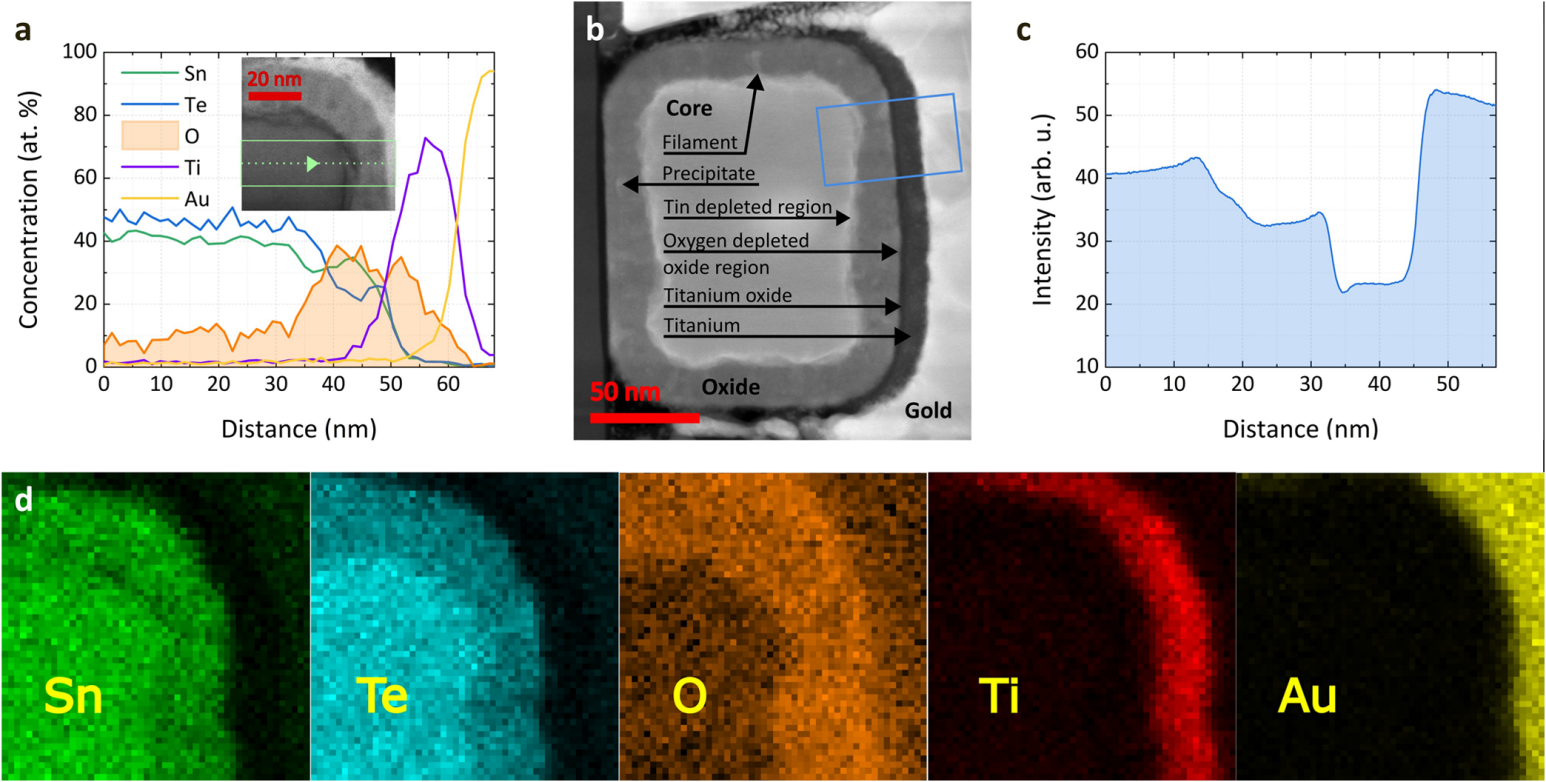
(a) EDS profiles of the elemental composition in the cross-section integrated along the green rectangle (shown in the inset) taken from PVD-grown NW coated on one side with Ti/Au bilayer. The zone axis is [100]. (b) HAADF-STEM image of the PVD-grown NW cross-section with indicated regions, precipitates, and filaments. (c) The intensity profile taken from the blue frame in panel (b). (d) Set of EDS elemental maps of the constituent elements of the metallized NW.

A detailed HAADF-STEM examination of the metal-coated sidewall region reveals additional features in the oxide skin compared to those in the nonmetallized nanowires (see Figure 4b). In addition to the previously observed precipitates, depletion of the oxide layer is visible as a (brighter; peak at the distance of about 32 nm in Figure 4c) region below the titanium layer. This is due to the absorption of a portion of the oxygen atoms in the titanium layer, which is revealed as an adjacent thin (darker; dip at the distance of about 34 nm in Figure 4c) region. This is compliant with EDS profiles, in a thin film, at the interface between the nanowire and metallization, where oxygen and titanium concentrations are comparable (see Figure 4a).

Figure 4d shows the EDS elemental maps to demonstrate their relative position and distribution of the elements, prepared in the same way as in the MBE case (Figure 3c,d). It can be seen that there are two rings as well: one depleted in tin and another one with an excess of tellurium (much weaker, below the former). Oxygen extends not only to the oxide outer region but also partially reaches the tin-depleted area. The atoms of the Ti/Au bilayer (metallization) do not penetrate the NW. These maps are consistent with the profiles obtained by the EDS method (Figure 4a).

The results presented above allow us to extract two important pieces of information. The first one is related to a phenomenon, unusual in nanowires, characterized by a radial (ring-like) redistribution of elements (see Figure 3). A similar observation is described by Shapiro et al. for SnTe/PbTe/SnO_2_ nanoparticles with diameters below 20 nm.^25^ The authors noticed migration of tin atoms to the surface, while lead atoms moved inward. This is known as the Kirkendall effect, in which the difference in the diffusion coefficients of elements plays a crucial role, and the mechanism is based on diffusion through vacancies. The second one is a specifically developed interface between the high-quality crystalline SnTe and the amorphous oxide skin (e.g., Figure 1e and f). Similar to the Kirkendall effect’s mechanism based on the anion exchange, filaments attached to the NW interior appear.^26^ However, unlike the nanoparticles presented in the aforementioned work, the formation of distinct voids inside the NW was not observed.

Since the Kirkendall effect describes the solid–solid interdiffusion, it cannot be adapted to a homogeneous material. The NWs studied in this work consist solely of binary SnTe. Thus, it should be stressed that the processes leading to the presented outcomes are primarily driven by oxidation. This is particularly pronounced in the NW fragments with sidewalls coated with gold (Figures 1c,d or 2b). In the protected parts, no oxide skin, no radial migration of elements, and thus no filaments are observed. SnTe oxidation has been described by Berchenko et al.^16^ where the authors present detailed studies using Raman spectroscopy, angle-resolved X-ray photoelectron spectroscopy, time-of-flight secondary ion mass spectroscopy, as well as Auger electron spectroscopy. The main conclusions depicted by the authors are consistent with our findings. Similar observations were reported for In-doped SnTe NW.^18^ Interestingly, upon closer inspection of the TEM images in this work, the filaments in the amorphous oxide skin, which are a fingerprint of the Kirkendall effect, can be identified (Figure 3a and b in ref 18). However, the authors did not mention such an observation.

Our results indicate that, at least for NWs widths greater than 50 nm, the oxidation kinetics is independent of the size of the NWs. This is in accordance with the findings presented by Anderson and Tracy.^26^ However, it should be noted that growth conditions, and thus the surface quality, have a strong influence on the oxidation process.

The oxidation mechanism proceeds in the two main paths. The initial stage is based on the Cabrera-Mott mechanism, originally proposed for the oxidation of metals.^27^ Recent modifications, which consider the spherical geometry of the nanoparticles and changes in the volume, make this approach adaptable to such structures.^28^ This is the trigger for the second stage—the Kirkendall effect. The flow of the process is shown schematically in Figure 5a. Gaseous oxygen from the air forms reactive oxygen species (ROS) by successive reduction and dissociation processes at the surface. The main donors are cationic redox centers. According to a review by Anpo et al.,^29^ the resulting oxygen ions are classified as suprafacial (adsorbed), intrafacial (surface-embedded), or mixed. Consequently, ROS accumulates a negative charge on the surface. Despite a comparable Pauling electronegativity value for tin and tellurium, it is mainly the former, as a cation, that interacts with oxygen on the surface.

**Figure 5:**
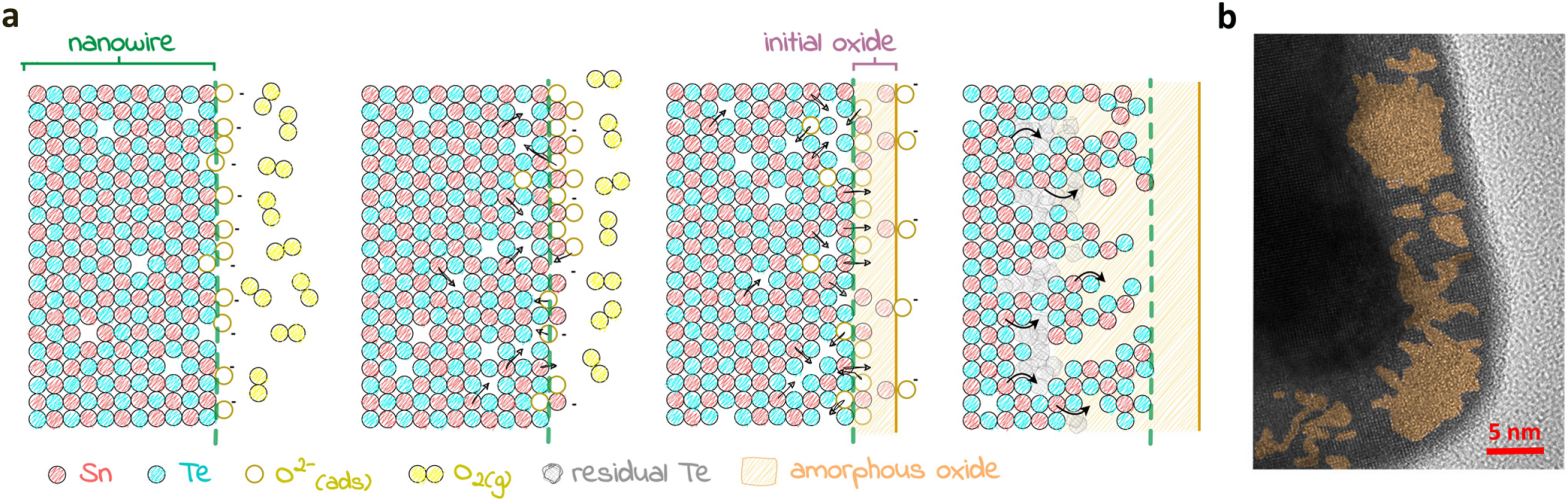
(a) Scheme of the oxidation mechanism in the Cabrera-Mott model, which initiates the interdiffusion of Sn^2+^ and O^2–^ ions. The last stage depicts the formation of filaments that enable tin migration along the boundary with the amorphous phase. The dashed green vertical line indicates the initial width of the nanowire. The gray color represents the area with excess amorphous tellurium. The black arrows show possible diffusion paths. (b) HR-TEM image corresponding to the last stage. Areas of the amorphous phase (mainly SnO_*x*_) are marked in orange.

Two possible diffusion mechanisms are considered. The first one is the interstitial mechanism, which is important for the diffusion of small atoms (in this case, oxygen) through the lattice of larger, less mobile atoms. For this reason, this type of diffusion is an implausible way for matrix elements to migrate. The second mechanism is diffusion through vacancies. As described by Mehrer, this is most likely in cases involving the Kirkendall effect.^30^ Due to the lower energy barrier, that mechanism is more probable than direct exchange or the ring mechanism, observed in the amorphous phase. This follows the relation between diffusion rate and vacancy density.^31^ In undoped IV–VI compounds, hole concentration can reach 10^21^ cm^–3^ (in SnTe).^32^ Density functional theory (DFT) calculations for SnTe show that the formation of native point defects in Sn-rich and Sn-poor regimes is associated with cation vacancies. They act as acceptors with the lowest possible formation energy of charged defects, including antisites.^33^ An important aspect to note is the size effect related to low-dimensional structures. As first-principles calculations reveal, point defects in the relaxation process are localized on the surface of the NW (as more stable) rather than internally.^34^

The outward migration of tin increases the concentration of cationic vacancies below the surface. Simultaneously, oxygen moves in the opposite direction. Migration of these two elements contributes not only to the formation of oxides below the surface, but also to their building up on the surface of the newly formed oxide skin, which is noticeable as NW swelling (Figure 2b). This is an important consequence of the relation between diffusion coefficients (*D*_Sn_ > *D*_O_ > *D*_Te_).^25^ Due to the supersaturation of Sn vacancies in the region showing trace amounts of oxygen, the anionic sublattice collapses. This has been classified as a significant but nonlocalized tellurium fraction (refs 16 or 18), and in our samples it is observed as a specific ring (see Figure 3).

In literature, the migration of atoms in the Kirkendall effect is described as movement along grain boundaries (surface diffusion).^35^ In our case, the outward migration of tin entails the inward migration of vacancies. The organized pathways of the vacancies, similar to the “caterpillar effect”, lead to the successive separation of nonoxygen-penetrated regions in the crystal structure, forming filaments. Since the vacancy movement is chaotic, the shape of the formed filaments is irregular, as in a ginger root. This is evident in sufficiently thick oxide skins. The filaments are surrounded by an amorphous phase, which affects their crystal structure. They exhibit a grainy structure along their entire length. Analysis shows twisting and tilting of the crystallographic orientation of these grains relative to the NW interior. The measured maximum twist increases linearly with the distance from the crystalline NW interior and saturates within ±5° at a distance of approximately 5 nm. We associate such twisting with effects provoked by the ring diffusion, which is possible at the interface with the surrounding amorphous phase. Whereas, the tilt (up to 7°) is attributed to the relaxation of stresses accumulated in the crystal structure of the filament, particularly at the constriction points.

As a result, the filaments exhibit a domain crystal structure along their entire length. More detailed information on the filaments is provided in Supporting Information Part 2. A general observation is that the linear density and length of the filaments strongly depend on the surface and structural quality of the NW. It should be noted that filaments are the main channels for the ion exchange in such a system, and this will continue until the original NW material is completely oxidized. Alternatively, the process can be stopped by physically breaking the filaments connecting the sources of the two elements being replaced.

When the Kirkendall effect is already initiated in the oxidation process, the metallization deposition does not significantly alter the internal structure of the nanowire. However, while the matrix elements (Sn or Te) do not migrate across the nanowire/metallization interface, oxygen diffuses beyond the preexisting oxide film, penetrating the titanium layer. Specifically, a few-nanometer-thick amorphous layer strongly saturated with oxygen (O:Ti ≈ 1:1) is created in the immediate area of this interface. Thus, it adds another component to the electronic structure of this system.

## Conclusions

We have studied the migration process of elements in tin telluride nanowires using TEM investigations. The semiconductor compound SnTe is recognized as a topological crystalline insulator, where surface states play a crucial role. The nanowires were grown in two modes: autocatalytic and gold-assisted. The use of two different growth techniques (MBE and PVD) shows the importance of surface quality in the oxidation processes of NWs exposed to ambient conditions. The negative charge accumulated on the surface during the oxidation (Cabrera-Mott mechanism), which is necessary to initiate the Kirkendall effect, occurs more easily on rougher surfaces with a higher density of surface vacancies.

Key observations, apart from the formation of an amorphous oxide skin (SnO_*x*_, typically semi-insulating), which poses a challenge in electron transport measurements, reveal the segregation of elements in the subsurface structure of the nanowire. This leads to the formation of pseudolayers toward the center of the NW structure, while the interior of the nanowire remains unchanged.

This process is based on the difference in diffusion coefficients of individual elements and is described as the Kirkendall effect. The main mechanisms involved in this case are the outward diffusion of tin through vacancies and the inward interstitial diffusion of oxygen. The tellurium ions, being the least mobile in this system, do not spread substantially and collapse into their sublattice due to the high concentration of tin vacancies. This creates a highly enriched Te layer between the crystalline SnTe and the amorphous outer region, which consists mainly of SnO_*x*_. The filaments carved out of the crystalline matrix during the interdiffusion process, characteristic of this effect, connect the NW to the outer oxide skin, ensuring ion exchange.

In the case of Au-catalyzed nanowires, thin films of Au in the form of a ferrule are observed on the sidewalls, protecting the material from oxidation. Such a coating is located below the nanodroplet and extends over a length of several dozen nanometers. The oxidized nanowires were coated with a bilayer of Ti/Au, a metallization commonly used for electrical contacts. Elemental analysis showed that titanium absorbs some of the oxygen from the preexisting oxide skin. This creates an additional amorphous area with a chemical composition similar to titanium monoxide at the interface.

Exposure of a sample to conditions other than an ultrahigh vacuum environment can lead to the adsorption of atoms or molecules, consequently, to modification of the surface layer. Cleaning processes such as ion etching (e.g., in hydrogen plasma) or sputtering with noble ions (e.g., Ar^+^) may prove insufficient to remove the effects of previous chemical modifications and reduce the resulting surface roughness. It is worth noting that, to date, IV–VI compounds have not been thoroughly investigated in this context. A natural way to protect nanowires from the Kirkendall effect is to eliminate the adsorption of ions that can diffuse into the structure. Coating the surface with a layer of noble metal (e.g., Au) effectively prevents oxidation processes but affects the physical properties of the system, in particular, electron transport. For this reason, a rational solution is to use core–shell structures in which the core of IV–VI material is covered with a shell of a II–VI compound (typically cadmium-based). This type of coating can effectively limit the diffusion of oxygen into the nanowire, contributing to the stabilization of its chemical, structural, and electronic properties.

## Supplementary Material



## Notes

Fabrication of electric contacts to SnTe nanowires; magnetoconductivity measurements at *T* = 4.2 K and *B* = ±1 T; characteristics of collection angles in an HAADF detector; spectrum images processing; crystalline filament twisting; influence of Au ferrule on PDV-grown nanowire; and estimation of nanowire volume change (PDF)
