# Supplementary material for: Oxidation-Induced Anisotropic Subsurface Segregation in SnTe Nanowires
Source: Chem Mater. 2026 Jul 28;38(15):7570–8. doi: 10.1021/acs.chemmater.5c03523 (PMC13471734; doi:10.1021/acs.chemmater.5c03523)
Supplement: Supplementary file 1 [file cm5c03523_si_001.pdf]

Supplementary Information

for

"Oxidation-induced anisotropic subsurface segregation in SnTe nanowires"

Dorota Janaszko[1,*], Piotr Dziawa[1,2], Jakub Polaczyński[2], Anna Kaleta[1], Bogusława Kurowska[1], Marta Bilska[1,3], Jędrzej Korczak[1,2], Aleksandr Kazakov[2], Tomasz Wojciechowski[2], Jakub Turczyński[1], Jerzy Dąbrowski[1], Janusz Sadowski[1,4], and Sławomir Kret[1]

[1]Institute of Physics, Polish Academy of Sciences, Al. Lotników 32/46, PL-02668 Warsaw, Poland

[2]International Research Center MagTop, Institute of Physics, Polish Academy of Sciences, Al. Lotników 32/46, PL-02668 Warsaw, Poland

[3]Institute of Experimental Physics, Faculty of Physics, University of Warsaw, ul. Pasteura 5, PL-02093 Warsaw, Poland

[4]Ensemble3 Center of Excellence, ul. Wólczyńska 133, PL-01919 Warsaw, Poland

*Email: janaszko@ifpan.edu.pl

Part 1

To study magneto-transport properties of single SnTe nanowires, we have applied a standard procedure for contact fabrication, consisting of e-beam lithography, metal evaporation, and lift-off. Contacted wires were measured using a home-made set-up with a He-cooled 8 T superconducting magnet. Details are given below.

Fabrication of electric contacts

Nanowires grown by both methods were transferred on commercially available SiO_2 /Si (100) substrates covered with a home-made grid of markers [1] (a "chip", shown in Figure S1). Markers enable the precise determination of the position of a single nanowire on the chip surface. The transfer was performed manually by scratching as-grown nanowires against the surface of a chip. Nanowires were then mapped using SEM operated at 5 kV. SEM images were used to design electric contacts connecting selected nanowires with large contact pads in `KLayout` software. Figure S2 shows an example of the nanowire transferred to a chip. Contacts were defined using a one-step electron beam lithography (EBL) process. A 450 nm thick layer of positive resist, PMMA 950 K dissolved in anisole (AR-P 672.06, AllResist) was deposited at 4000 rpm and

baked at 170 °C for 15 min. Exposure was performed using a Zeiss Auriga Scanning Electron Microscope coupled with an ELPHY Plus nanolithography kit from Raith. Operating voltage of 30 kV was used with apertures 20 μm or 10 μm, corresponding to the current of 115 pA or 30 pA, respectively, depending on the size of the exposed structure; the dose used was 400 $\mu C/cm^2$. Exposed resist was developed in MIBK:IPA 1:3 solution for 60 s and rinsed in IPA for 30 s. After development, samples were cleaned using oxygen plasma and rinsed in a 1% HCl solution to remove residuals of the resist and tin oxide covering the nanowires' surface. 5 nm Ti/140 nm Au contacts were deposited in a thermal evaporator from PREVAC, operating at 2×10^{-6} mbar base pressure. Lift-off in acetone was performed to remove excess metal.

Transport measurements

After contact fabrication, chips were mounted on standard 8-pin DIP sockets, and 25 μm thick Au wire was attached to contact pads using a wedge wire bonder, leaving one end of the wire floating to be later connected to socket pins using Ag paste. The chips were grounded during both wire bonding and the connection of the wire to the socket pins. Magneto-transport experiments were performed using a standard 8 T superconducting magnet cooled with liquid He, with a home-made sample probe. The low-frequency lock-in technique, with $I_{AC} < 100$ nA (usually 10 nA), was used to collect the signal. We note that those measurements were hindered by the high fragility of nanowires, which are prone to electrical discharges.

Results of magnetotransport measurements

Despite the fragility of fabricated nanodevices, we have collected a signal from several PVD-grown SnTe nanowires. An example of 2D magnetoconductivity of such a sample, symmetrized relative to $B = 0$, is presented in Figure S3. The overall signal shows parabolic field dependence with a cusp-like feature near zero field. Although transport results for SnTe nanowires are sparse [2, 3], our observation agrees with previously published data. The low-field cusp-like structure is typical for weak antilocalization (WAL) described by the Hikami-Larkin-Nagaoka (HLN) formula, which for the materials with strong spin-orbit coupling takes the following form [4]:

$$\Delta\sigma = \frac{\alpha}{2\pi}\left[\ln\left(\frac{\hbar}{4el_\phi^2 B}\right) - \Psi\left(\frac{\hbar}{4el_\phi^2 B} + \frac{1}{2}\right)\right]$$

where e is the electron charge, l_ϕ is a phase coherence length, α is a factor related to the total number of conducting channels, and Ψ is a digamma function. We have fitted the formula above to our experimental data in the field range $B = \pm 1$ T. The result is shown as a red curve in Figure S3, and more details are shown in Figure S4. We obtained $l_\phi = 314$ nm and $\alpha = 1.33$.

Observation of weak localization is in agreement with the presence of Topological Surface States, expected for a topological insulator. However, direct comparison of our results with literature data is not possible – WAL was observed in $Pb_{1-x}Sn_xTe$ nanowires [2], while other experiments on pure SnTe samples have only shown parabolic magnetoresistance [3].

Table S1: Collection of angular range (inner and outer radii, in mrad) dependence on camera length (in mm) for the HAADF detector installed in the TEM Titan used in these studies [6].

Detector no.	4	5	7	9
Camera length	73	91	145	230
Inner radius	80.3	65.5	40.4	24.9
Outer radius	196.6	207.3	228.5	152.1

Part 2

Characteristics of the collection angles in high-angle-annular dark-field detector

The relation between camera length and collection angle can vary for different microscopes. For the Titan Cube 80-300 equipped with an image corrector (installed at IF PAN), the collection angles of the high-angle-annular dark-field (HAADF) detector were determined in the works of A. Kaleta and coworkers [5, 6]. The ranges of collection angles and corresponding camera lengths used in the present study are summarized in Table S1. The ratio of inner angle to outer angle is limited by the active area of the scintillator and does not entirely depend on the length of the camera. For our equipment, this value is determined to be 6.1 ± 0.2. The procedure for measuring the inner collection angle is described in detail in Appendix A of the paper by LeBeau et al. [7]. The outer collection angle is obtained by multiplying the inner collection angle by a physical ratio measured for a given device. It should be noted that, due to the unfavorably smaller angular range of transferred diffraction than the outer detector, the cutoff angle of the microscope optics on scattered electrons has a significant impact on the camera lengths below 145 mm [8]. The above circumstances explain the limitations of the collection angle of the outer ADF detector, which results either from a lower cutoff value or from the limited surface area of the scintillator.

Processing of Data Cube spectrum image

The quantitative data were collected in the form of a three-dimensional spectrum image referred to as a "data cube". To determine the quantitative concentration of elements of interest in various areas, individual spectra from selected regions were integrated to obtain low-noise spectra. For example, in the case of the nanowire cross-section, the Sn(Lα) spectral lines were selected (Figure S5a). Using the "Windows mapping" function of SiTools, a two-dimensional intensity distribution of these lines was generated (Figure S5b).

The distribution clearly shows a dark ring about 5 nm thick surrounding the nanowire interior, where the intensity of the Sn(Lα) lines is reduced. To obtain a high signal-to-noise ratio from this particular region, individual spectra from selected pixels in the dark ring (red squares in Figure S5b) were integrated. In this way, quantification can be obtained from specific regions. The result is shown in Figure S5c.

In addition to the expected spectral lines of tin, tellurium, and oxygen, elements present in the lattice, namely copper and carbon, are visible. The latter also comes from hydrocarbon contamination. The spectrum also shows lines of residual gallium, which was used in the focused ion beam (FIB) to produce the cross-sectional specimens, as well as lines of silicon coming from the substrate (see Figure S5a).

The twist of the crystalline precipitates in the PVD-grown nanowires

While examining the PVD-grown nanowire cross-sections, some crystalline precipitates are noticed in the amorphous oxide layer. These are fragments of filaments, shaped by the Kirkendall process perpendicular to the lamellae. Those parts vary in shape and size (diameters from 4 nm to as much as 12 nm), with most being round. They have the same crystal structure as the interior of the nanowire. However, they show twisting in both clockwise and counterclockwise directions, also visible in TEM and Fast Fourier Transform (FFT) images (Figure S6).

The absolute maximum twist angle reaches up to 9 degrees (Figure S7). The structural evolution of the filaments has not been the subject of particular interest in the literature to date, and their twist has not yet been described. There is no clear relation between twist and distance from the nanowire interior. Initially, near the nanowire interior, the maximum absolute twist increases linearly with distance. However, most of the measured twist values are within the range of ± 5 degrees. This suggests that there is some limitation to the angle, which may be due to steric, geometric, or structural factors. We have not investigated this behavior because it is not the subject of our current research. Nevertheless, we believe that the occurrence of such a twist is interesting to study.

Observation of a whole individual filament that has been cut through horizontally allows us to see the progress of twisting along the filament under examination. The evolution of the twist of two single filaments is presented in Figure S8a and S8b. In addition to twisting, the filaments show deviation from the crystal lattice planes in the nanowire interior (see Figure S8c). It indicates that during evolution, the filament also turns in directions other than rotational motion. The reason and mechanism of this behavior remain unresolved and require in-situ investigation.

Influence of Au ferrule on the PVD-grown nanowire

The metallization of the oxidized nanowire does not significantly affect the oxides that have already formed. However, diffusion of a certain amount of oxygen into the titanium layer is observed (see Fig. 4a in the main text). The most important effect of the metallization is the deactivation of the Cabrera-Mott mechanism, i.e., blocking access to the oxygen reservoir. A similar effect is observed in Au-catalyzed nanowires with the gold ferrule. In Fig. S9, we present such a difference for areas of nanowires whose walls are covered with ferrule and those without such a coating. Additionally, we can observe clear signs of an increase in the total width of the nanowires (crystalline SnTe and amorphous oxide) caused by the growth of oxides (see Fig. S10).

The original volume of the nanowire

Using the simple calculations, it is possible to attempt to reconstruct the original volume of the nanowire based on the excess tellurium in the tin-depleted region (area marked with green in Figure S11). The tellurium-rich region is created by the escape of tin ions toward the nanowire surface. Such a tin-depleted region is saturated with vacancies, located in the cation sublattice. This leads to the disintegration and collapse of the crystal structure, thereby changing the physical volume. This is also manifested as a 1.3 nm shift in profiles between the maximum and minimum of Te and Sn content, respectively. The excess tellurium, marked on the profile, would enable the formation of a crystal lattice together with Sn, which would widen the current width of the nanowire by 1 nm on each side. This value approximately agrees with the mentioned shift in composition. However, we did not do *in situ* observations of how the anion sublattice collapses in

terms of crystallographic directions and other parameters. We only observe the effects of this phenomenon. Taking into account the above observations, the results are as follows:

- Concerning the interior of the nanowire and tellurium-rich region, the width shrank by 4.5%.
- Migration of tin, followed by the formation of tin oxides, caused the nanowires to expand by 17.6%.

References

(1) Lähnemann, J.; Ajay, A.; Den Hertog, M. I.; Monroy, E. Near-Infrared Intersubband Photodetection in GaN/AlN Nanowires. *Nano Letters* **2017**, *17*, 6954–6960.

(2) Safdar, M.; Wang, Q.; Wang, Z.; Zhan, X.; Xu, K.; Wang, F.; Mirza, M.; He, J. Weak Antilocalization Effect of Topological Crystalline Insulator Pb1–xSnxTe Nanowires with Tunable Composition and Distinct 100 Facets. *Nano Letters* **2015**, *15*, 2485–2490.

(3) Liu, P.; Han, H. J.; Wei, J.; Hynek, D.; Hart, J. L.; Han, M. G.; Trimble, C. J.; Williams, J.; Zhu, Y.; Cha, J. J. Synthesis of Narrow SnTe Nanowires Using Alloy Nanoparticles. *ACS Applied Electronic Materials* **2020**, *3*, 184–191.

(4) Hikami, S.; Larkin, A. I.; Nagaoka, Y. Spin-Orbit Interaction and Magnetoresistance in the Two Dimensional Random System. *Progress of Theoretical Physics* **1980**, *63*, 707–710.

(5) Kaleta, A.; Kret, S.; Gas, K.; Kurowska, B.; Kryvyi, S. B.; Rutkowski, B.; Szwacki, N. G.; Sawicki, M.; Sadowski, J. Enhanced Ferromagnetism in Cylindrically Confined MnAs Nanocrystals Embedded in Wurtzite GaAs Nanowire Shells. *Nano Letters* **2019**, *19*, 7324–7333.

(6) Kaleta, A. Structure and Properties of Magnetic Hybrid Nanowires Based on Transmission Electron Microscopy of High Spatial and Energetic Resolution, Ph.D. Thesis, Institute of Physics, Polish Academy of Sciences, 2022.

(7) LeBeau, J. M.; Stemmer, S. Experimental Quantification of Annular Dark-Field Images in Scanning Transmission Electron Microscopy. *Ultramicroscopy* **2008**, *108*, 1653–1658.

(8) Krause, F. F.; Schowalter, M.; Grieb, T.; Müller-Caspary, K.; Mehrtens, T.; Rosenauer, A. Effects of Instrument Imperfections on Quantitative Scanning Transmission Electron Microscopy. *Ultramicroscopy* **2016**, *161*, 146–160.

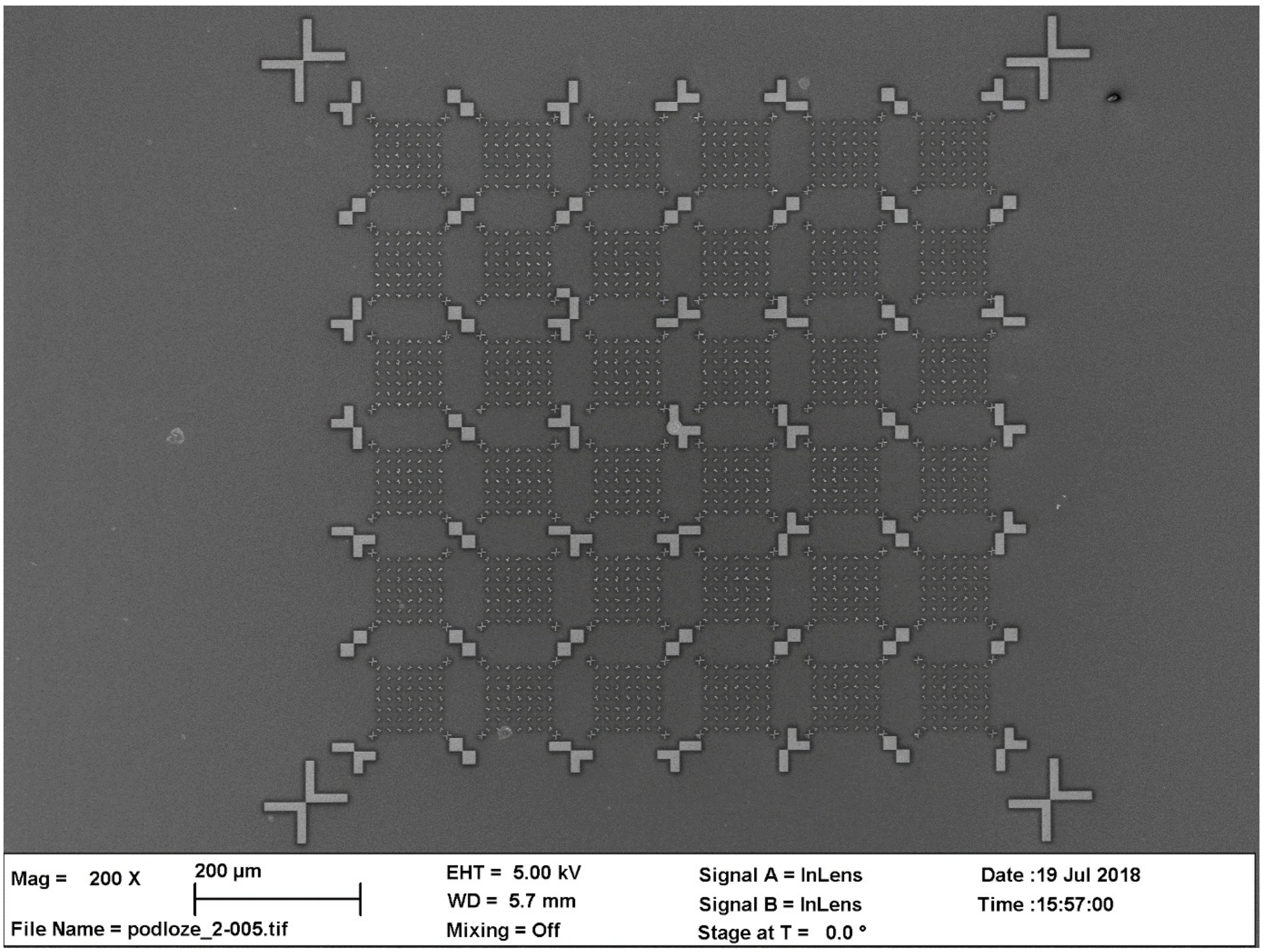

Figure S1: SEM image of a chip used for fabrication of electric contacts to single SnTe nanowires.

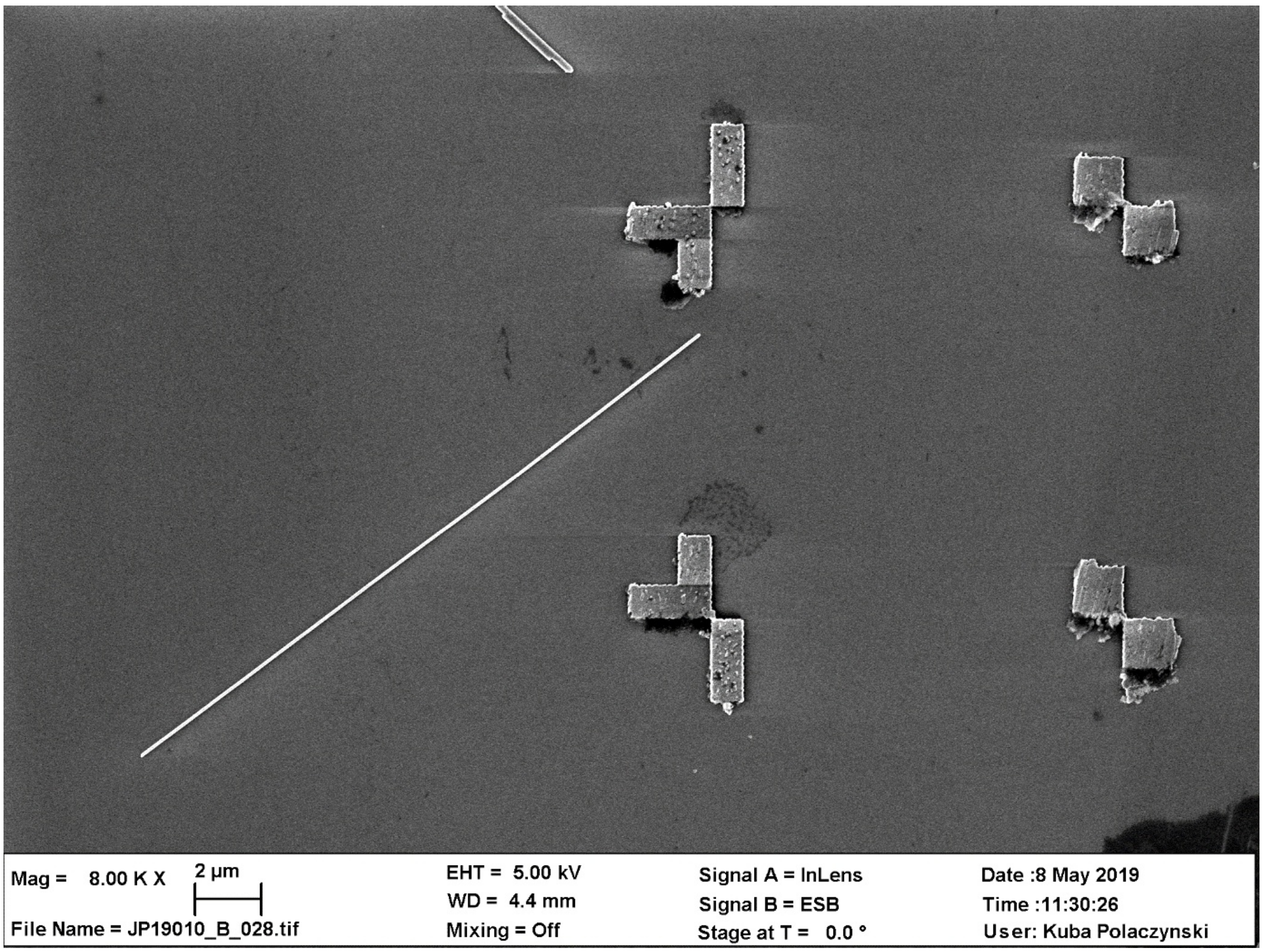

Figure S2: SEM image of a SnTe nanowire transferred to the chip.

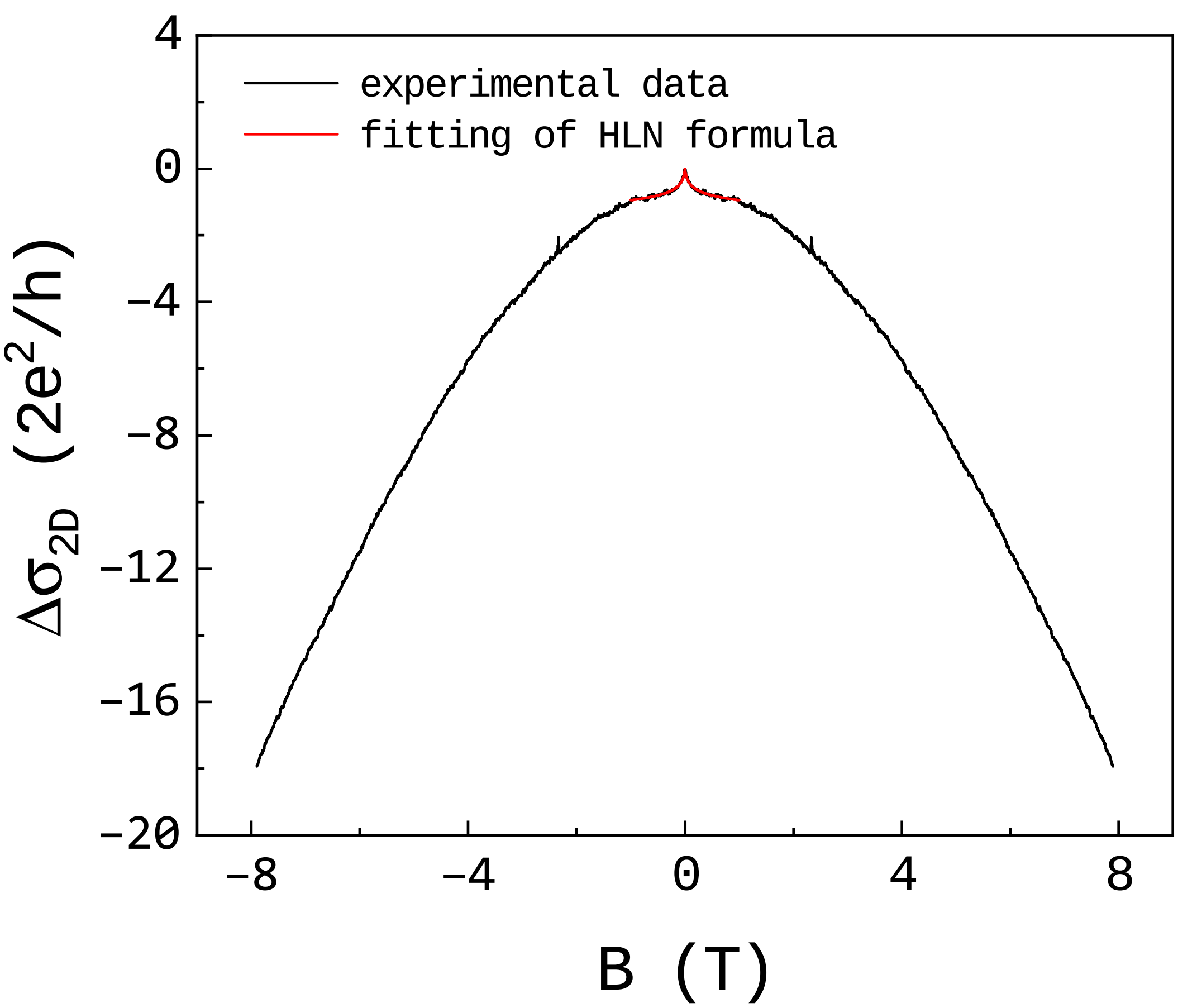

Figure S3: Experimental magnetoconductivity measured for PVD-grown SnTe nanowire at $T = 4.2$ K (black curve). The red curve shows the result of fitting the 2D HLN formula for fields $B = \pm 1$ T.

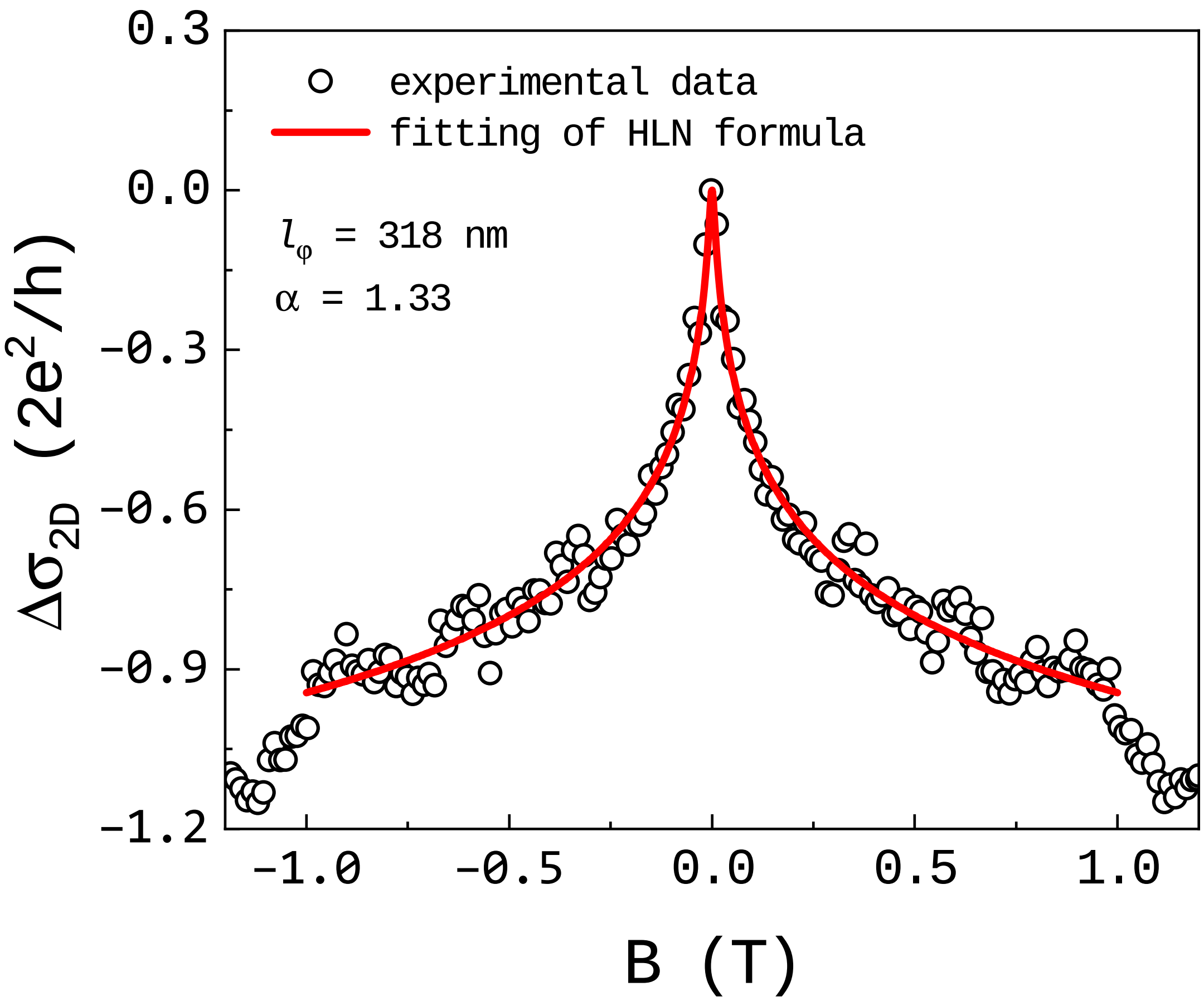

Figure S4: Magnified low-field magnetoconductivity from Figure S3. The red curve shows the fitting of the HLN formula to our experimental data in the field range $B = \pm 1$ T.

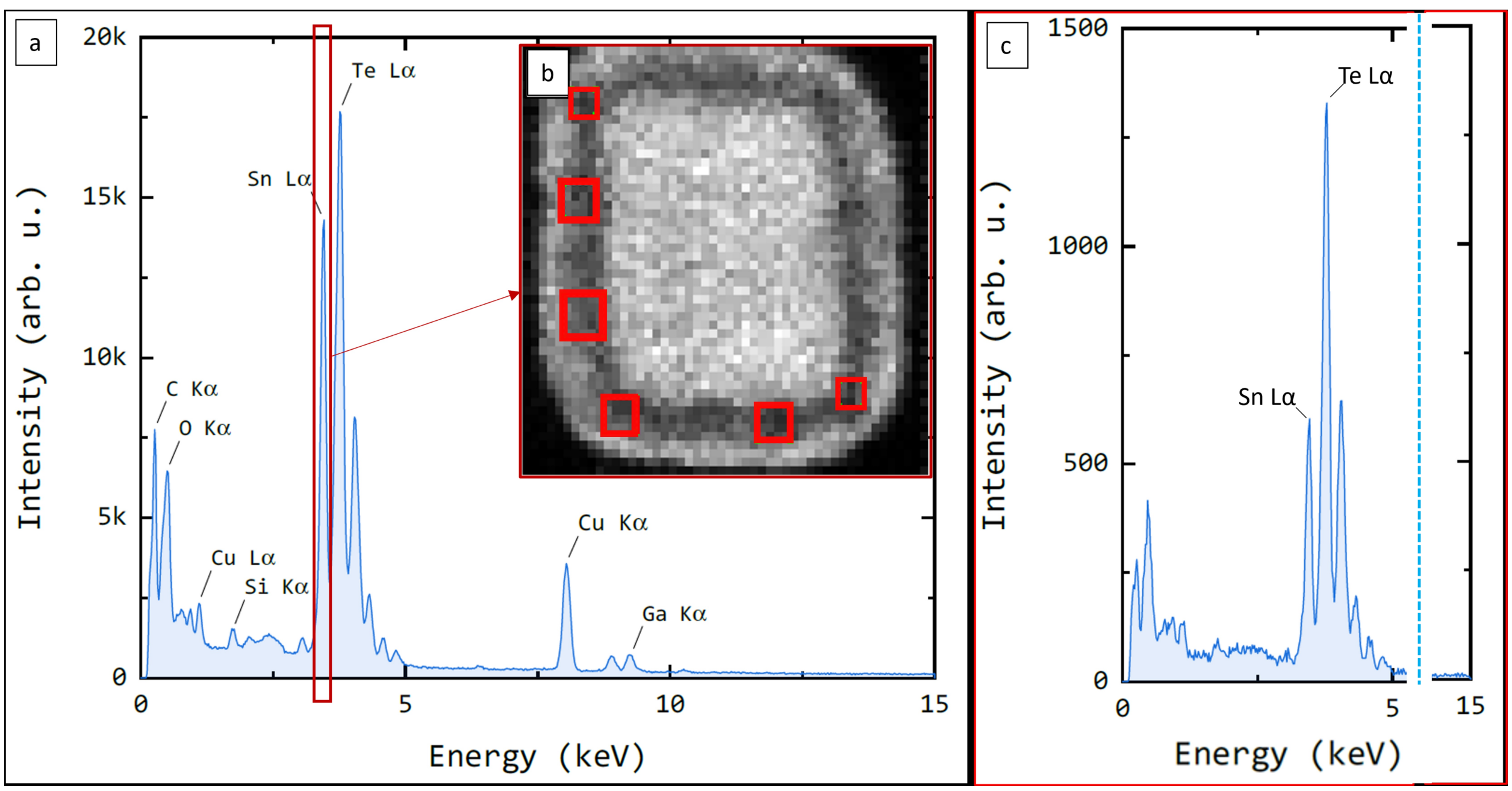

Figure S5: Methodology of data processing: (a) Spectrum from the whole data cube with summed signal intensity. (b) Mapped spectrum image obtained from Sn L spectral line. (c) Integrated spectrum image obtained only from areas marked by red squares in panel (b).

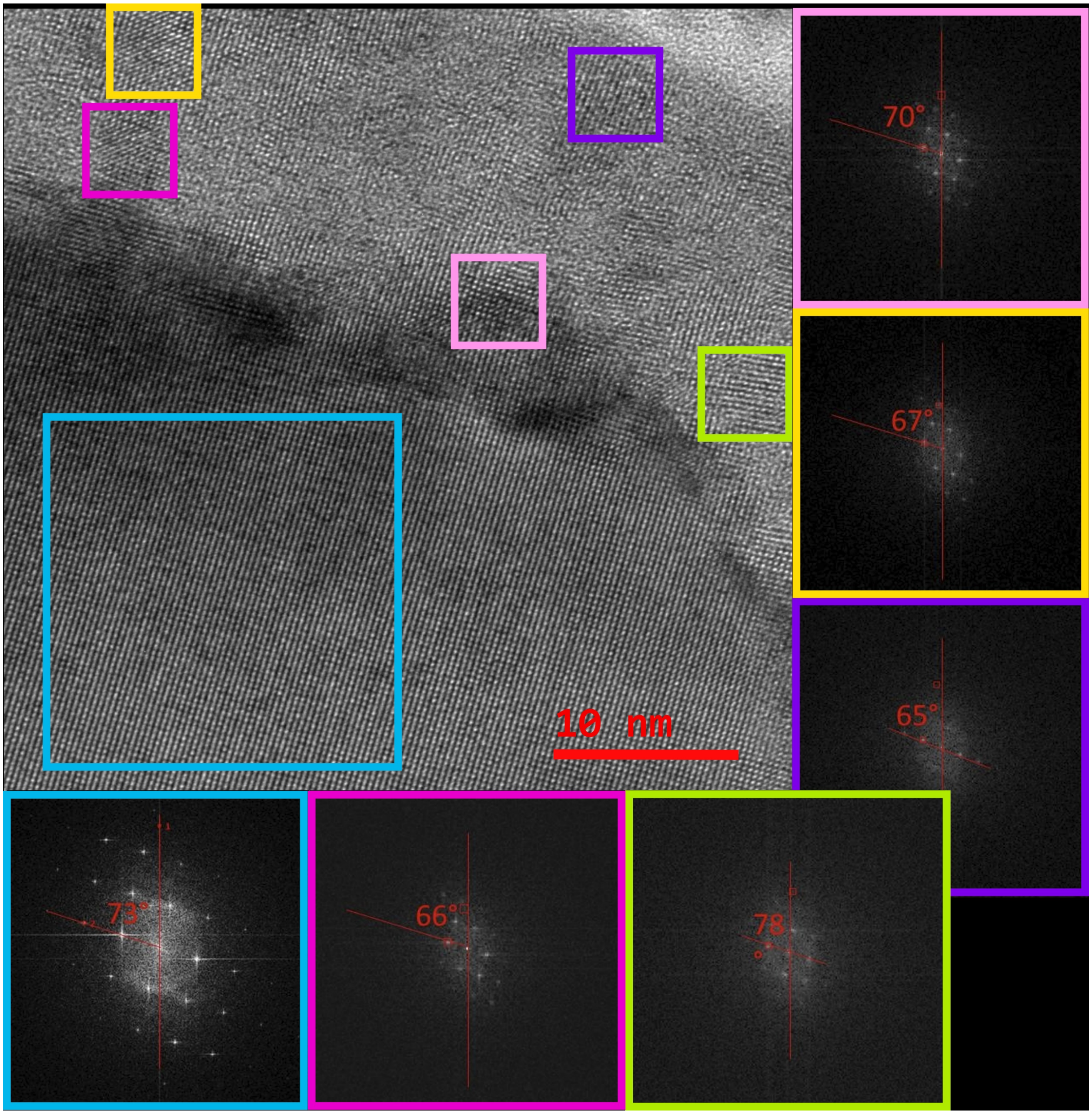

Figure S6: HRTEM image of the nanowire cross-section and extracted FFT patterns. The inner region of the nanowire serves as a reference (large blue box). Smaller defined regions and corresponding FFT images are marked with the same colors. Differences in orientation are shown (labeled with specific angles).

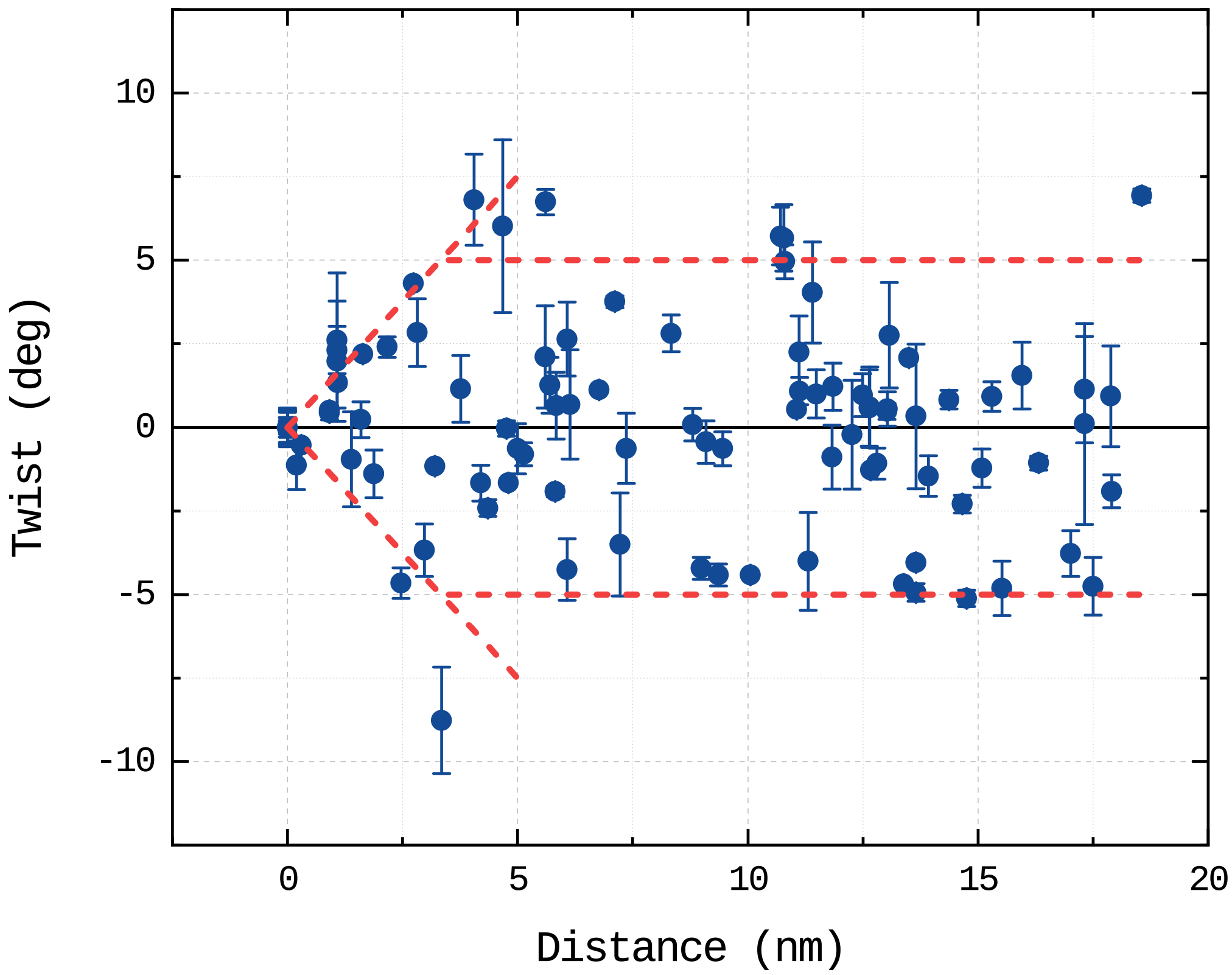

Figure S7: The relation between twist and distance from the nanowire interior for filament cross-sections.

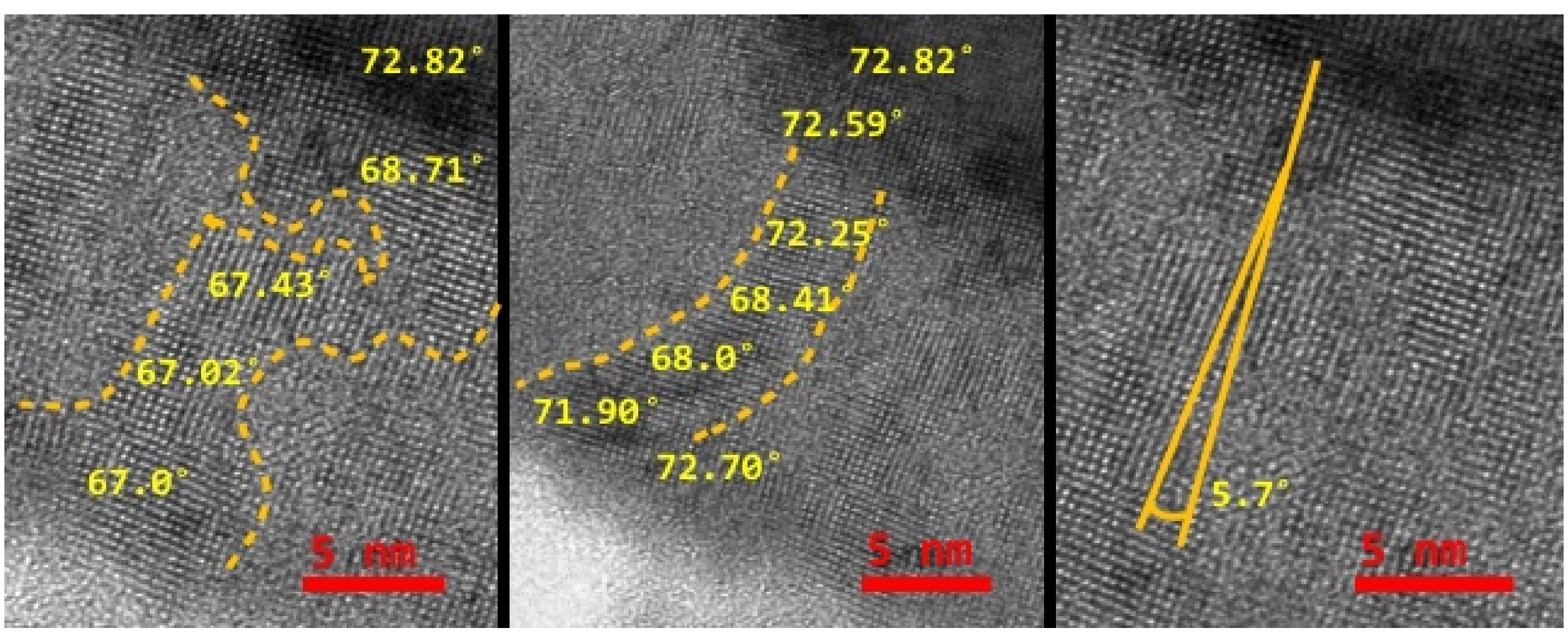

Figure S8: HRTEM images of cross-sectioned PVD-grown nanowire with filaments marked by orange dashed lines. The angles are measured between the same (111) reflex on FFT and the central line, where 72.82° is the angle obtained for the nanowire interior. The right panel shows the deviation of the lattice plane in the filament from that in the nanowire interior.

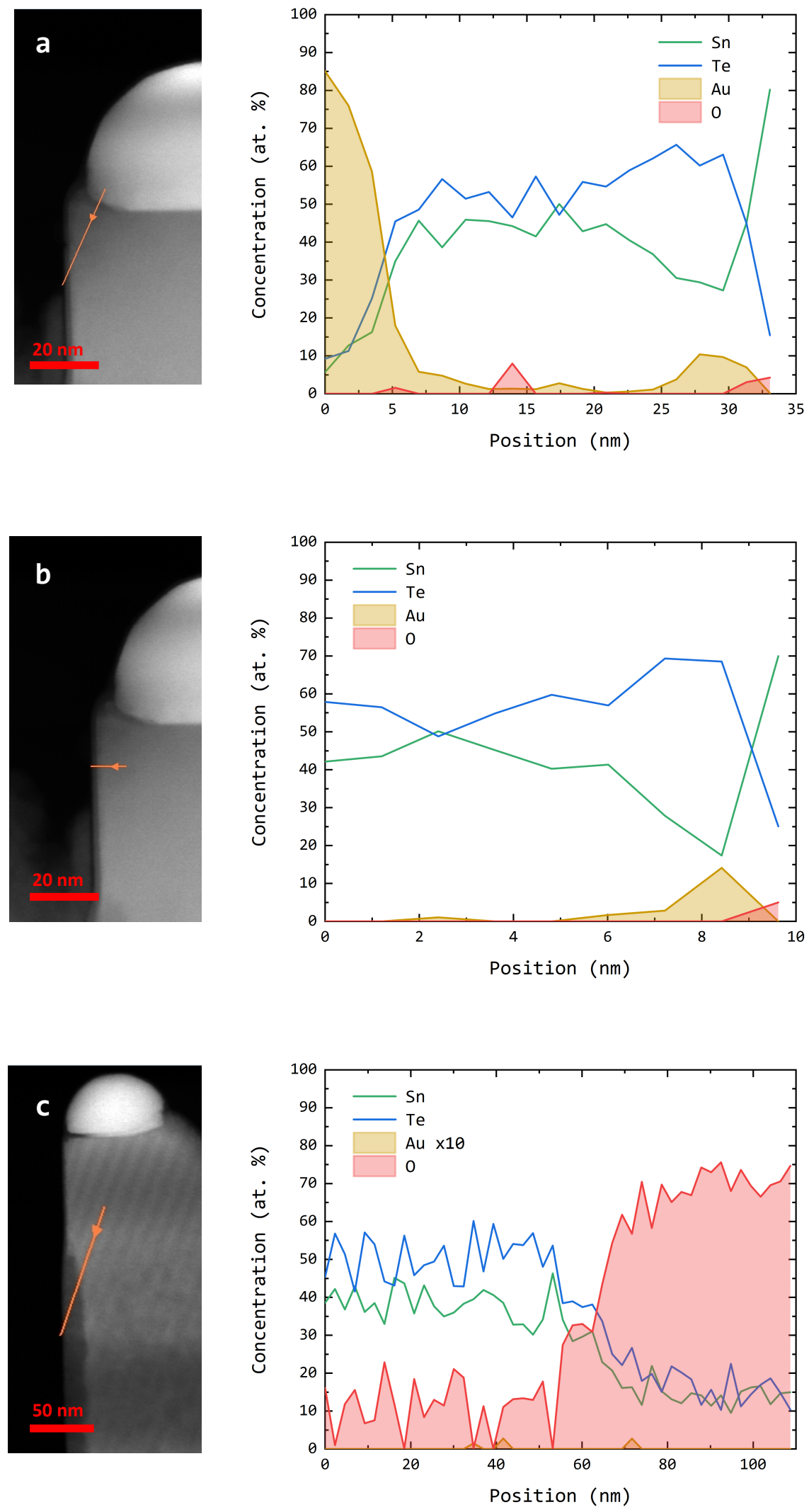

Figure S9: An example of the influence of an Au ferrule on nanowire oxidation. (a-c) HAADF-STEM images and corresponding profiles taken at three different places of the same nanowire. Fringes observed in image (c) are moiré patterns.

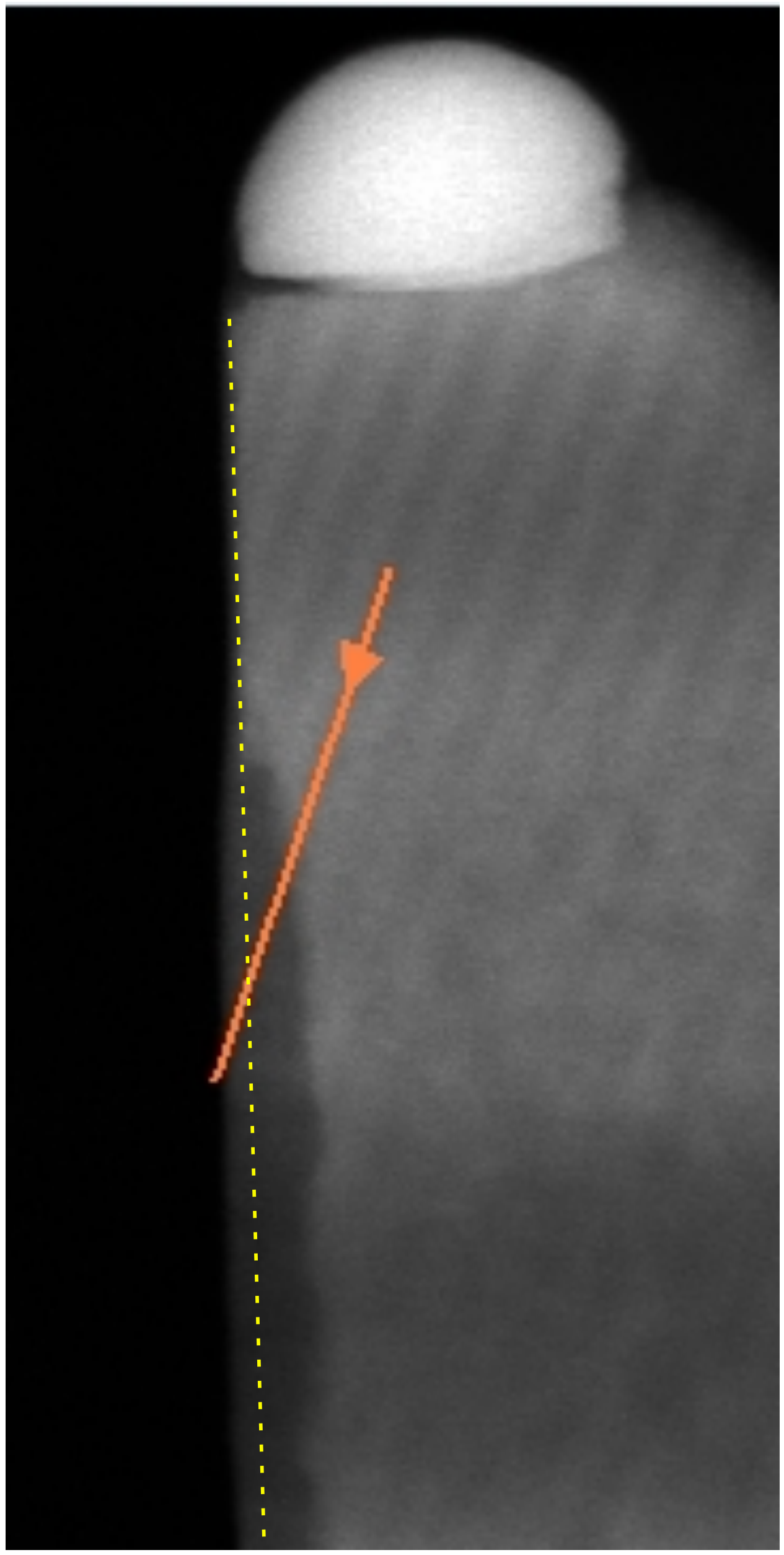

Figure S10: Image from panel Fig. 9c. It provides evidence of change in the total width (oxides /darker/ and crystalline SnTe /brighter/) of the unprotected part of the nanowire as a result of oxidation. Note the dashed line along the edge of the unoxidized nanowire with a thin film of gold.

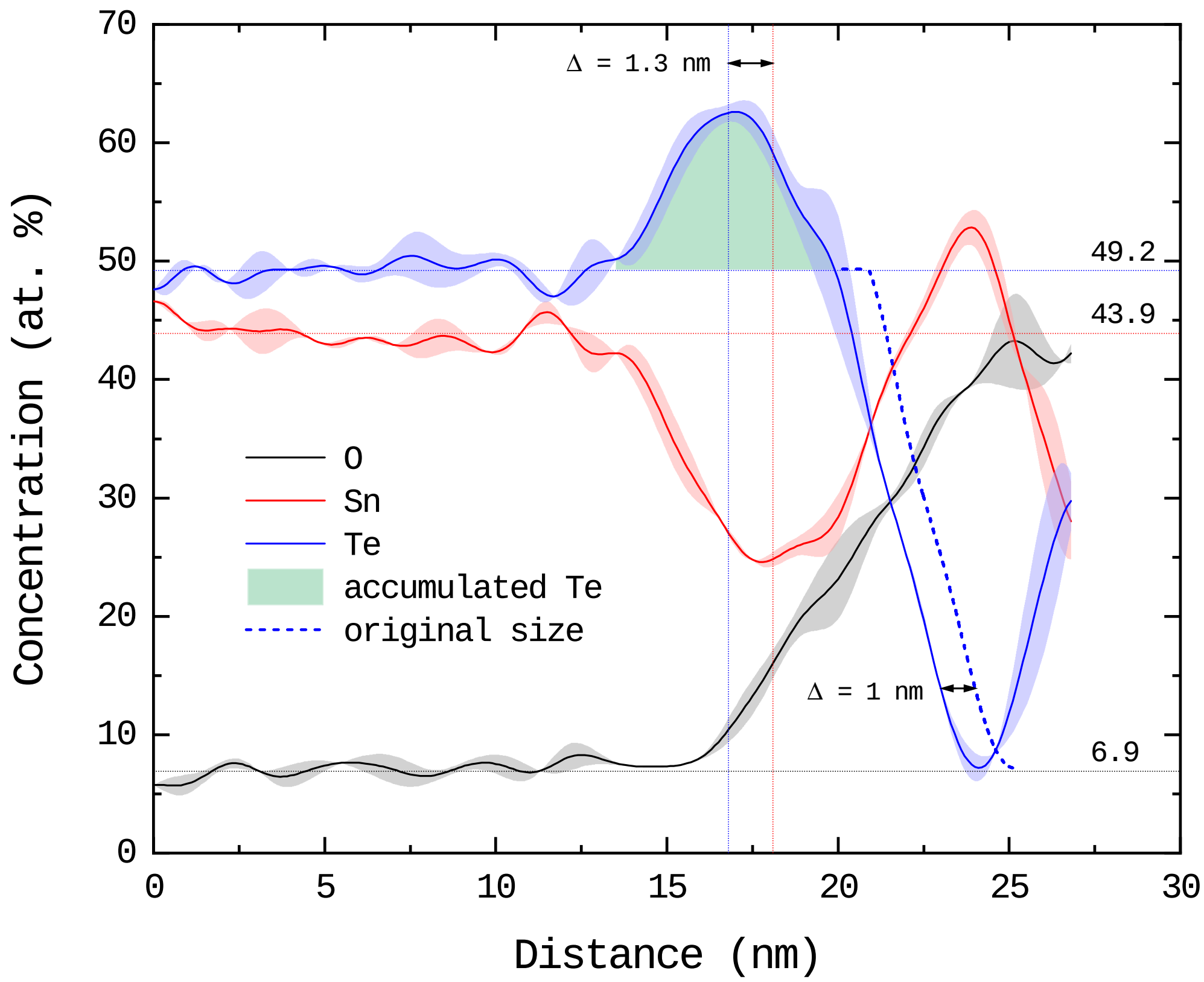

Figure S11: EDX profile of the cross-section of a nanowire grown by MBE, averaged relative to the center point of the cross-section (distance equal to 0). The shaded areas around the solid lines indicate uncertainty. The dotted blue line indicates the original size of the nanowire before oxidation and the associated collapse of the crystal structure just below the oxide skin.
